# Prognostic significance of ST6GalNAc-1 expression in patients with non-metastatic clear cell renal cell carcinoma

**DOI:** 10.18632/oncotarget.11258

**Published:** 2016-08-12

**Authors:** Qi Bai, Li Liu, Wei Xi, Jiajun Wang, Yu Xia, Yang Qu, Ying Xiong, Qilai Long, Jiejie Xu, Jianming Guo

**Affiliations:** ^1^ Department of Urology, Zhongshan Hospital, Fudan University, Shanghai 200032, China; ^2^ Department of Biochemistry and Molecular Biology, School of Basic Medical Sciences, Fudan University, Shanghai 200032, China

**Keywords:** clear cell renal cell carcinoma, a-GalNAc a-2,6-sialyltransferase 1, nomogram, overall survival, recurrence free survival

## Abstract

**Background:**

Sialyltransferase ST6GalNAc-1 is highly expressed in tumor cells and associated with tumor aggressiveness and poor prognosis. In the present study, we aimed to investigate the clinical and prognostic significance of sialyltransferase ST6GalNAc-1 in patients with non-metastatic ccRCC.

**Results:**

High expression of ST6GalNAc-1 in tumor tissue was an independent prognostic factor for overall survival (*p*<0.001) and recurrence free survival (*p*<0.001) in multivariate analysis. The nomograms could give better prediction for overall survival and recurrence free survival in ccRCC patients.

**Methods:**

264 patients diagnosed with non-metastatic clear cell renal cell carcinoma were enrolled in the present study. Immunohistochemical staining was performed on tissue microarrays to evaluate the intratumoral ST6GalNAc-1 expression. Kaplan-Meier method and Cox proportional hazard model were applied to assess the prognostic value of ST6GalNAc-1. Nomograms were generated to refine individual risk stratification in ccRCC patients.

**Conclusion:**

ST6GalNAc-1 was an independent adverse prognostic factor for both overall survival and recurrence free survival in patients with non-metastatic ccRCC.

## INTRODUCTION

Renal cell carcinoma (RCC) is the most common solid lesion within kidney and accounts for 2-3% of all cancer in adults [1]. There has been 209,000 new cases and 102,000 deaths worldwide per year, and the majority of RCCs (70%) are classified as clear cell renal cell carcinoma (ccRCC) [2]. Of the patients diagnosed with RCC, 20%-30% are presented with metastatic RCC (mRCC), and 30% of the patients with localized disease will ultimately develop metastases even after the curative surgeries [3]. In the metastatic setting, mRCC remains largely incurable. Although the overall survival of patients with mRCC has improved substantially in the past decade owing to targeted therapy agents, complete responses were reported in only 1-3% of patients and vast majority of patients still die of their disease [4–6]. These finds may highlight the need for continuous exploration of RCC biology and novel approaches to RCC management.

In recent years, glycosylation is considered as a key regulator of malignant transformation and pathways relating to cancer progression [7]. Sialylation is one of the most-widely occurring cancer-associated changes, as sialylated carbohydrates may mediate pathophysiological events during the various steps of tumor progression [8]. One principal mechanism underlying the altered expression of sialylated glycan can be attributed to under- or overexpression of glycosyltransferase [9]. In malignant cells, α-GalNAc α-2,6-sialyltransferase 1 (ST6GalNAc-1), which adds sialic acid in an α-2,6 linkage to a serine or threonine residue, is responsible for carcinogenesis in types of tumor [10–12]. Thus, we speculated that ST6GalNAc-1 might be a potential prognosticator in ccRCC.

To verify the clinical and prognostic importance of sialyltransferase ST6GalNAc-1 in ccRCC, we assessed the ST6GalNAc-1 expression by immunohistochemistry in non-metastatic ccRCC tissues. Moreover, we built two nomograms, which integrated with independent prognostic parameters refine individual risk stratification in non-metastatic ccRCC patients.

## RESULTS

### ST6GalNAc-1 expression in ccRCC

**Figure 1:**
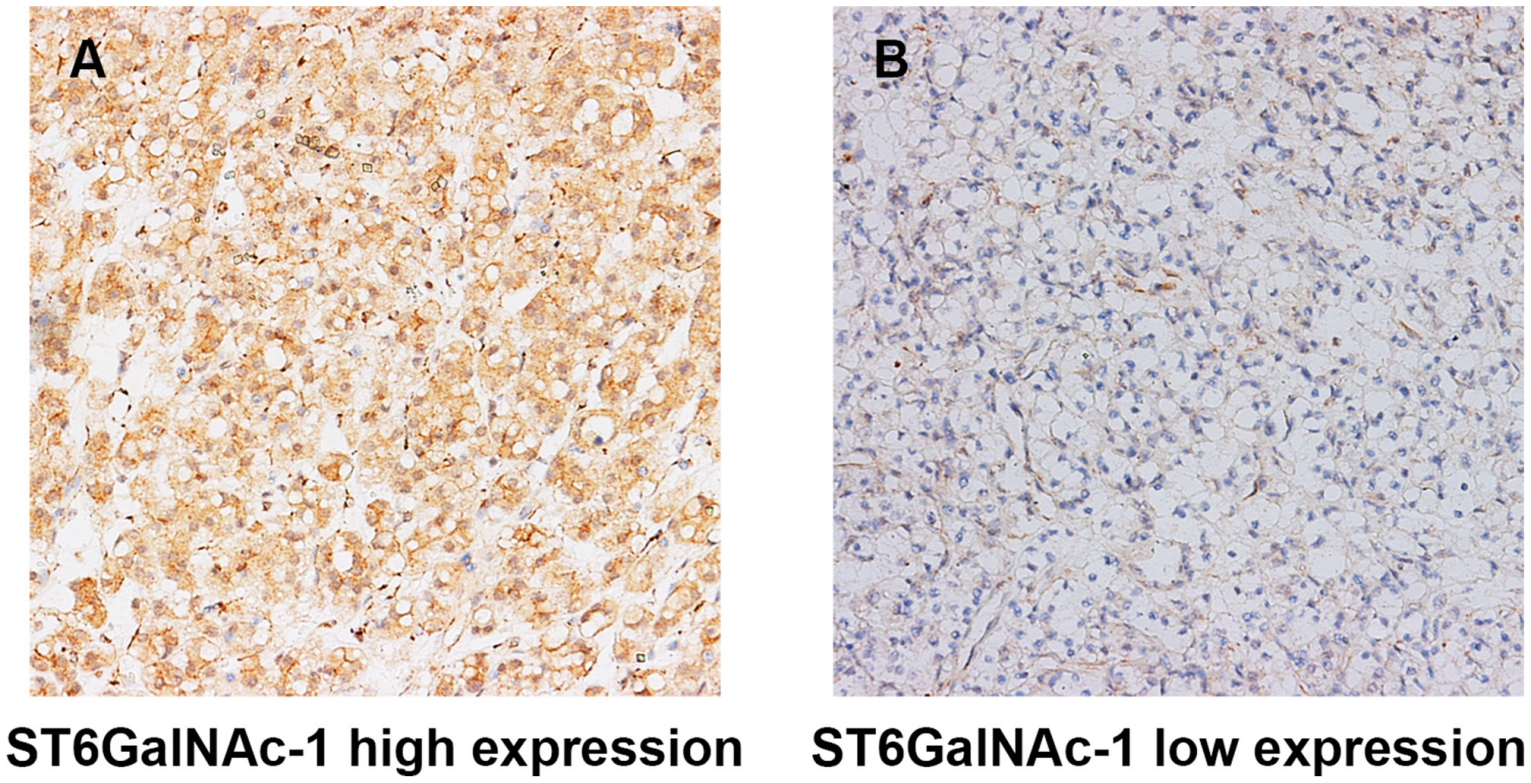
Representative photographs of ST6GalNAc-1 immunostaining High ST6GalNAc-1 expression in tumor tissue **A.** Low ST6GalNAc-1 expression in tumor tissue **B.** Original magnification: ×200.

**Figure 2:**
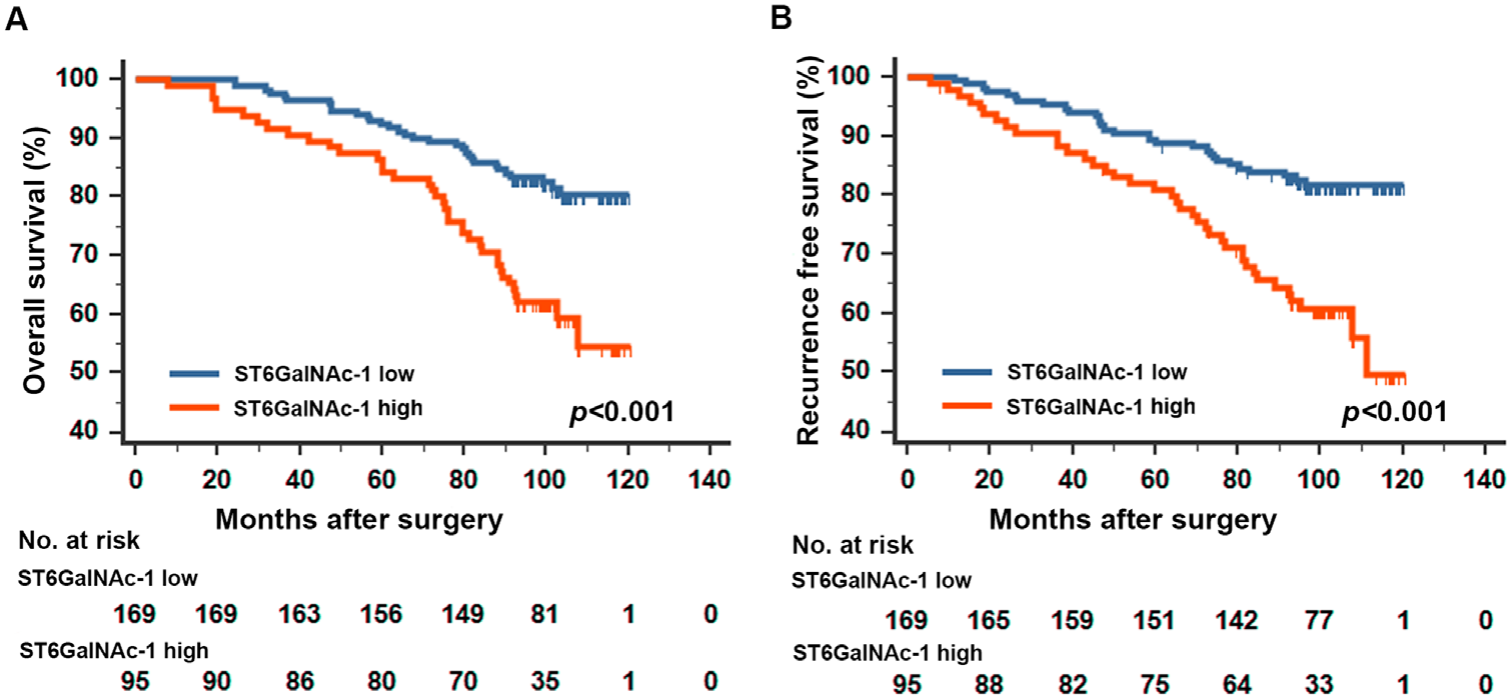
Kaplan-Meier analyses for overall survival and recurrence free survival of patients with ccRCC according to ST6GalNAc-1 expression Overall survival according to ST6GalNAc-1expression in non-metastatic ccRCC patients **A.** recurrence free survival according to ST6GalNAc-1expression in non-metastatic ccRCC patients **B.** *p*-value was calculated by Log rank test, *p*<0.05 was regarded as statistically significant.

In normal kidney tissue, the positive staining of ST6GalNAc-1 could be detected in glomerulus and nephric tubule, and the expression of ST6GalNAc-1 was homogeneous between different tissue (Figure 1A). In ccRCC, the positive staining predominantly appeared in the cytoplasm in tumor tissue (Figure 1B, 1C). We analyzed a total of 264 patients in the present study. The clinical characteristics and their correlation with ST6GalNAc-1 are exhibited in Table 1. The median follow-up time was 99 (range 2.63-120.47) months. The survival curves showed that patients with high ST6GalNAc-1 expression tend to have significantly dismal outcome for overall survival (OS) (*p*<0.001, Figure 2A) and recurrence free survival (RFS) (*p*<0.001, Figure 2B) than those with low ST6GalNAc-1 expression patients.

**Table 1:** Correlation between ST6GalNAc-1 expression and clinical characteristics in localized ccRCC patients

Variables	All patients		ST6GalNac-1 expression		
	No.	%	Low	High	p^*^
Age at surgery. yr					0.421
≤55	133	50.4	82	51	
>55	131	49.6	87	44	
Gender					0.156
Female	83	31.4	48	35	
Male	181	68.6	121	60	
ECOG PS					0.506
0	188	71.2	118	70	
≥1	76	28.8	51	25	
Surgery					0.117
Partial nephrectomy	26	9.8	13	13	
Radical nephrectomy	238	90.2	156	82	
Tumor size, cm					0.954
≤4.0	155	58.7	99	56	
>4.0	109	41.3	70	39	
Pathological T stage					0.421
pT1	171	64.8	110	61	
pT2	22	8.3	15	7	
pT3	67	25.4	43	24	
pT4	4	1.5	1	3	
Fuhrman nuclear grade					0.427
1	28	10.6	21	7	
2	195	73.9	123	72	
3	38	14.4	24	14	
4	3	1.1	1	2	
Necrosis					0.541
Absent	226	85.6	143	83	
Present	38	14.4	26	12	

Abbreviations: ECOG PS: Eastern Cooperative Oncology Group performance status. ccRCC: clear cell renal cell carcinoma.

*χ^2^ test was performed. p<0.05 was regard as statistically significant.

### Extension of prognostic models with ST6GalNAc-1

**Figure 3:**
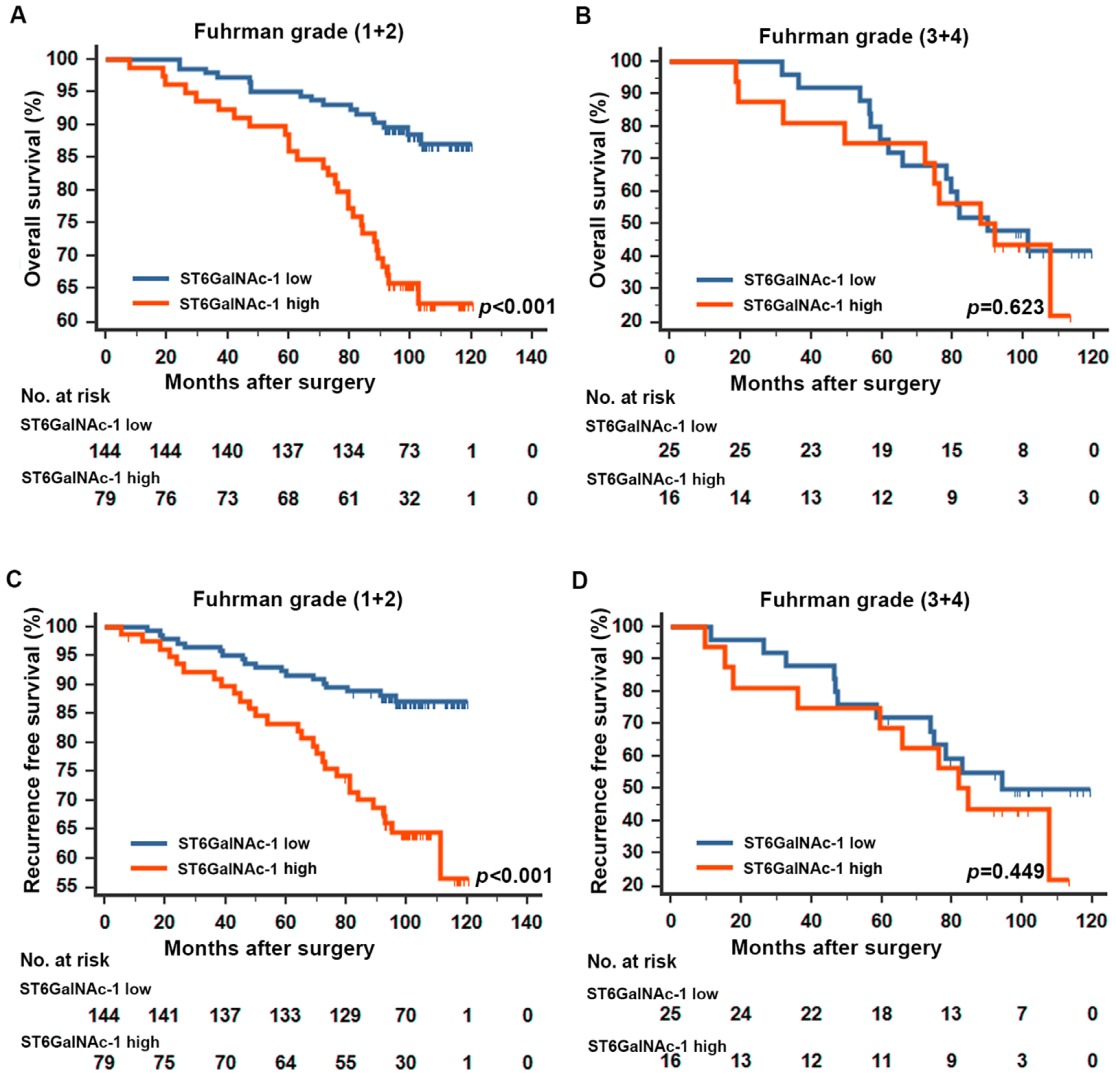
Kaplan-Meier analyses for overall survival and recurrence free survival of patients in Fuhrman grade subgroups Overall survival for patients in the Fuhrman grade (1+2) group **A.**, and (3+4) group **B.** according to ST6GalNAc-1 expression; recurrence free survival for patients in the Fuhrman grade (1+2) group **C.**, and (3+4) group **D.** according to ST6GalNAc-1 expression; p-value was calculated by Log rank test, p<0.05 was regarded as statistically significant.

Based on the results above, we further performed a subgroup analysis by Fuhrman grade in the study. The prediction value of ST6GalNAc-1 was only restricted in patients within Fuhrman grade (1+2) group (Figure 3A, 3C). ST6GalNAc-1 failed to further distinguish clinical outcome in patients within Fuhrman grade (3+4) group. Furthermore, we analyzed the ST6GalNAc-1 prognostic value in different SSIGN/Leibovich subgroups. The results showed ST6GalNAc-1’s prediction value on both OS and RFS in SSIGN/Leibovich subgroup analyses (Figure S1). ST6GalNAc-1 also proved to be an independent prognostic factor in different SSIGN/Leibovich subgroups (Figure S2).

### Multivariate cox regression analyses

Multivariate Cox regression models were applied to investigate the prognostic impact of ST6GalNAc-1 on ccRCC (Table 2). The results showed patients with higher ST6GalNAc-1 expression had a significantly reduced OS (*p*<0.001) and RFS (*p*<0.001) compared with their counterparts. It was also confirmed that pT stage (*p*<0.001), Fuhrman grade (*p*<0.001), and Necrosis (*p*=0.001) were independent prognostic factors for OS, and pT stage (*p*<0.001), Fuhrman grade (*p*<0.001) and necrosis (*p*=0.001) were independent prognostic factors for RFS in ccRCC.

**Table 2:** Univariate and multivariate cox regression analyses for overall survival and recurrence free survival in localized ccRCC patients

Variables	Univariate analysis		Multivariate analysis	
	HR(95% CI)	p^*^	HR(95% CI)	p^*^
Overall survival				
pT stage		**<0.001**		**<0.001**
pT2 *vs* pT1	3.70 (1.78-7.68)	**<0.001**	4.12(1.56-10.89)	**0.004**
pT3 *vs* pT1	3.94(2.33-6.67)	**<0.001**	4.44(2.45-8.04)	**<0.001**
pT4 *vs* pT1	10.01(3.01-33.29)	**<0.001**	10.81(2.98-39.29)	**<0.001**
Fuhrman grade		**<0.001**		**<0.001**
2 *vs* 1	1.53(0.55-4.27)	0.419	1.15(0.40-3.26)	0.798
3 *vs* 1	5.35(1.83-15.60)	**0.002**	3.72(1.24-11.17)	**0.019**
4 *vs* 1	7.39(1.65-33.08)	**0.009**	9.10(1.91-43.31)	**0.006**
Necrosis (present *vs* absent)	3.30(1.97-5.51)	**<0.001**	2.52(1.44-4.44)	**0.001**
Tumor size (continuous, cm)	1.19(1.09-1.29)	**<0.001**	0.99(0.88-1.12)	0.858
ECOG PS (≥1 *vs* 0)	1.303(0.79-2.151)	0.300	-	-
ST6GalNAc-1 (high *vs* low)	2.51(1.56-4.04)	**<0.001**	2.74(1.66-4.52)	**<0.001**
Recurrence-free survival				
pT stage		**<0.001**		**<0.001**
pT2 *vs* pT1	4.23(2.09-8.56)	**<0.001**	4.91(1.88-12.82)	**0.001**
pT3 *vs* pT1	3.22(1.88-5.52)	**<0.001**	4.37(2.67-8.06)	**<0.001**
pT4 *vs* pT1	17.87(6.10-52.33)	**<0.001**	20.22(6.29-65.02)	**<0.001**
Fuhrman grade		**<0.001**		**<0.001**
2 *vs* 1	1.20(0.47-3.03)	0.704	0.84(0.32-2.18)	0.721
3 *vs* 1	3.60(1.34-9.66)	**0.011**	2.85(1.02-8.00)	**0.047**
4 *vs* 1	5.89(1.41-24.79)	**0.015**	6.78(1.52-30.23)	**0.012**
Necrosis (present *vs* absent)	3.15(1.87-5.31)	**<0.001**	2.47(1.39-4.38)	**0.002**
Tumor size (continuous, cm)	1.19(1.09-1.29)	**<0.001**	0.99(0.88-1.12)	0.859
ECOG PS (≥1 *vs* 0)	1.19(0.71-1.99)	0.511	-	-
ST6GalNAc-1 (high *vs* low)	2.61(1.62-4.22)	**<0.001**	2.88(1.73-4.79)	**<0.001**

Abbreviations: ECOG PS: Eastern Cooperative Oncology Group performance status; HR: hazard ratio; CI: confidence interval; ccRCC: clear cell renal cell carcinoma.

*Data obtained from the Cox proportional hazards model; *p* <0.05 was regard as statistically significant.

### Nomogram for predicting overall survival and recurrence free survival in ccRCC

**Figure 4:**
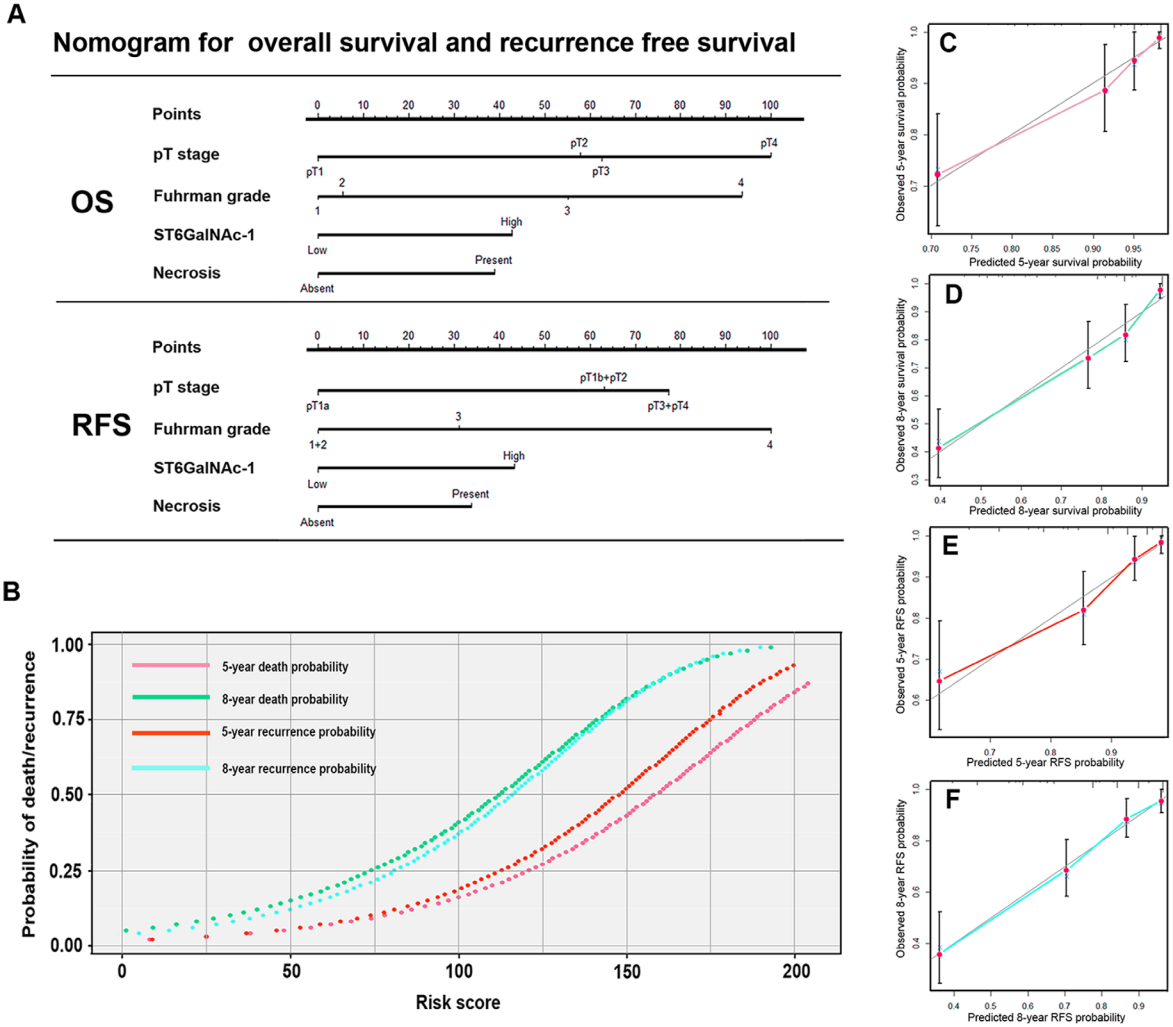
Nomogram for predicting 5- and 8-year overall survival and recurrence free survival in patients with ccRCC Nomogram for predicting 5- and 8- year OS and RFS, higher total point indicated a more adverse outcome probability **A.** parameter gram to calculate the probability of death or recurrence **B.** Calibration plot for nomogram predicted and observed 5-year overall survival rate **C.** and 8-year overall survival rate **D.** Calibration plot for nomogram predicted and observed 5-year recurrence free survival rate **E.** and 8-year recurrence free survival rate **F.** Line of dashes: ideal model, vertical bars: 95% confident interval.

We built two nomograms to predict OS and RFS (Figure 4A, 4B) at 5 and 8 years after nephrectomy. Total points were used as parameters to evaluate the clinical outcome, with higher point indicating a worse outcome probability. Calibration plots of the nomograms are shown for OS (Figure 4C, 4D) and RFS (Figure 4E, 4F) separately. The Harrell’s c-indices, were 0.800 (95%CI, 0.750-0.844) and 0.790 (95%CI 0.742-0.837) for OS and RFS respectively, higher than the combination of independent prognostic factors except ST6GalNAc-1, 0.762 (95%CI: 0.709-0.815) and 0.752 (95%CI: 0.702-0.801). Moreover, the Harrell’s c-indices of SSIGN and Leibovich scores were 0.734 (95%CI, 0.677-0.791) and 0.753(95%CI, 0.704-0.802), which indicates a better performance of the present nomogram.

## DISCUSSION

In the present study, we evaluated the prognostic importance of ST6GalNAc-1. The results revealed the dismal role of ST6GalNAc-1 and its impact on patients with ccRCC by survival analyses. Patients with higher level of ST6GalNAc-1 expression tend to have unfavorable clinical outcomes than the counterparts. Then, we found ST6GalNAc-1 could further stratify patients’ clinical outcome in Fuhrman grade (1+2) subgroup. The nomograms which integrated ST6GalNAc-1 with other prognostic parameters could serve as better prediction model for OS and RFS in ccRCC patients.

It is well established that glycosylation is one of the most common forms of posttranslational modifications. The most-widely occurring cancer-associated changes in glycosylation are sialylation, fucosylation, O-glyan truncation, and N- and O-linked glycan branching [9, 13]. Sialic acids are one of the most important monosaccharide being expressed as terminal sugars in several cell surface molecules. The sialylation of glycan can dramatically alter the behavior of cells as sialylated carbohydrates have important roles in cellular recognition, cell adhesion and cell signaling [14]. Abnormally high level of sialylated tumor associated carbohydrate antigens are frequently described at the surface of cancer cells. It is now well established that altered sialyltransferase activation contributes to the aberrant sialylation of glycan and expression of specific tumor-associated carbohydrates [8]. In malignant cells, ST6GalNAc-1is highly expressed and associated with carcinoma aggressiveness and poor prognosis. Hidenori Ozaki elucidated the roles of ST6GalNAc-1 by *in vivo* monitoring system in gastric cancer metastasis [10]. Tamura, F showed overexpression of ST6GalNAc-1 is associated with enhanced cell growth, infiltration, and migratory of MKN74 cells, and in peritoneal dissemination model, mice had longer survival time when treated with ST6GalNAc-1 siRNA, which suggesting ST6GalNAc-1 as a potential target for treatment of malignant disease [15]. The researches indicated ST6GalNAc-1 might be involved in tumor development including proliferation, migration, and invasion ability.

Recently, influence of ST6GalNAc-1 on malignant activities is widely recognized through its regulation of Tn and sTn antigens [16–18]. Altered Tn/sTn level associates with malignant activities including cell-cell/cell-ECM adhesion, cell migration, cell invasion and immunoregulation. In the early time, sTn level has been used as an independent predictor for cancer aggressiveness and metastatic in both colorectal [19] and ovarian cancer [20]. Meantime, aberrant sTn is commonly detected in a variety of carcinomas including colorectal [18, 21], gastric [22], ovarian [23], breast carcinomas [17], but rarely detected in normal tissue. S Julien found an increased tumorigenicity after transplantation of ST6GalNAc-1 transfected sTn-positive human breast cell line in to mice [11]. In addition, sTn has also been used as a target for cancer immunotherapy in preclinical and clinical studies [24, 25].

Generally speaking, it may be difficult to fully explain ST6GalNAc-1’s pro-tumor effects solely by the regulation of sTn synthesis. Recently, ST6GalNAc-1 has received attention for its ability to control Gal-1- and Gal-3-binding moieties on O-glycans which significantly impact the ferocity of cancer growth and metastasis [26]. Inhibition of ST6GaLNAc-1 could suppress STAT5b phosphorylation and then as result decreased IGF-1 expression, and then stimulate the dissemination of malignant cells [15]. In addition, Kim et al suggests the possibility that sialylation by ST6GalNAc-1 may affect sialic acid residues on gangliosides which is essential for the activation of JAK-STAT signaling [27]. In spite of all the possible biology functions of ST6GalNAc-1 in tumorigenicity, we should realize that mechanisms underlying the biology function of ST6GalNAc-1 are far from fully elucidation and merit further research.

Limitations of the present study are the retrospective design and only patients with non-metastatic disease are involved. The evaluation of ST6GalNAc-1 expression was mainly based on immunohistochemistry, which is kind of subjective compared with methods like Rini et al. did before [28]. A multicenter and prospective study is needed to validate the results in a larger population.

In conclusion, we have revealed that ST6GalNAc-1 expression is an independent prognostic factor in non-metastatic ccRCC by survival analyses. Patients with higher ST6GalNAc-1 expression are more likely to suffer unfavorable clinical outcome than the counterparts. Therefore, we have reason to believe that ST6GalNAc-1 might play a pivotal role in ccRCC progression.

## PATIENTS AND METHODS

The population based cohort study was conducted in 264 patients of non-metastatic ccRCC who underwent nephrectomy between Jan 2005 and Jun 2007 in Zhongshan Hospital, Fudan University (Shanghai, China). Clinical records and demographic characters include age, sex, tumor size, TNM stage, and ECOG PS were extracted from the database of the institution. The access to medical record was approved by related departments. All the patients underwent either nephron spare surgery or radical nephrectomy. Tumor stage and postoperative histopathological type was determined according to the 2010 AJCC TNM classification [29]. The inclusion criteria were as follows: (1) the histopathological type proved to be ccRCC, (2) all the individuals had no history of anticancer therapy before the nephrectomy, (3) had no history of other malignant disease before, and (4) patients with N1 or M1 tumors were excluded from the present study. All sections from nephrectomy samples were re-evaluated by a practiced pathologist to determine the Fuhrman grades, histology type, and presence of necrosis. If the histopathology was mostly necrosis (>80%) or the morphologic features represent a mixture type of ccRCC and other RCC type, samples were excluded from the present study.

Most patients underwent regular follow-up every 6 months or earlier for the first 2 years right after the nephrectomy and every 12 months thereafter. Clinical Research Ethics Committee of Zhongshan Hospital, Fudan University had approved the study with the approval number B2015-030 in Feb 2015. Written, informed consent was obtained from each individual enrolled in the study.

### Immunohistochemistry and evaluation

We performed immunohistochemistry staining on tissue microarrays (TMAs). The TMAs construction and immunohistochemistry protocol were described previously [30]. The primary antibody was ST6GalNAc-1 antibody (NBP1-87043). The staining intensity and extent was scored by two independent pathologists who were blind to the clinical outcomes. Fields were at x200 magnification and the intensity score was graded as 0 (negative), 1 (weak), 2 (moderate), and 3 (strong); the extent score was calculated by the percentage of the positive cells (0%-100%). The staining intensity and extent were then multiplied to generate the expression score ranging from 0 to 300. The score of 200 was selected as the cutoff point of high/low expression by the X-Tile software (Yale University School of Medicine, New Haven, CT, USA).

### Statistical analyses

χ^2^ test or Fisher’s exact method test were applied for assessing correlations between ST6GalNAc-1 expression and patients’ clinical characteristics. Survival curves were established using Kaplan-Meier method and statistical significance was calculated using log-rank test. Univariate and multivariate Cox proportional hazard models were used to test the impact of demographic characteristics, clinical features and ST6GalNAc-1 expression on overall survival and recurrence free survival. All statistical tests were two sided and considered significant at p <0.05 levels.

We used R software with “rms” package (R Foundation for Statistical Computing, Vienna, Austria) to generate the nomogram. Selection of the parameters in nomogram was based on statistical significance of multivariate analyses. We combined T stage T1b and T2 in nomogram due to the clinical similarity for metastases after radical nephrectomy. Calibration plots for 5- and 8- year OS and RFS were generated to explore the performance characteristics of the predictive model. Harrell’s concordance indices (c-indices) were used to measure the prognostic accuracy. All data analyses above were performed using SPSS version 19.0 (SPSS Inc., IL, Chicago, USA) and R software with “rms” package (R Foundation for Statistical Computing, Vienna, Austria).

## SUPPLEMENTARY MATERIALS FIGURES


