# Supplementary material for: Prognostic significance of ST6GalNAc-1 expression in patients with non-metastatic clear cell renal cell carcinoma
Source: Oncotarget. 2016 Aug 12;9(3):3112–20. doi: 10.18632/oncotarget.11258 (PMC5790450; doi:10.18632/oncotarget.11258)
Supplement: Supplementary file 1 [file oncotarget-09-3112-s001.pdf]

Prognostic significance of ST6GalNAc-1 expression in patients with non-metastatic clear cell renal cell carcinoma

SUPPLEMENTARY FIGURES

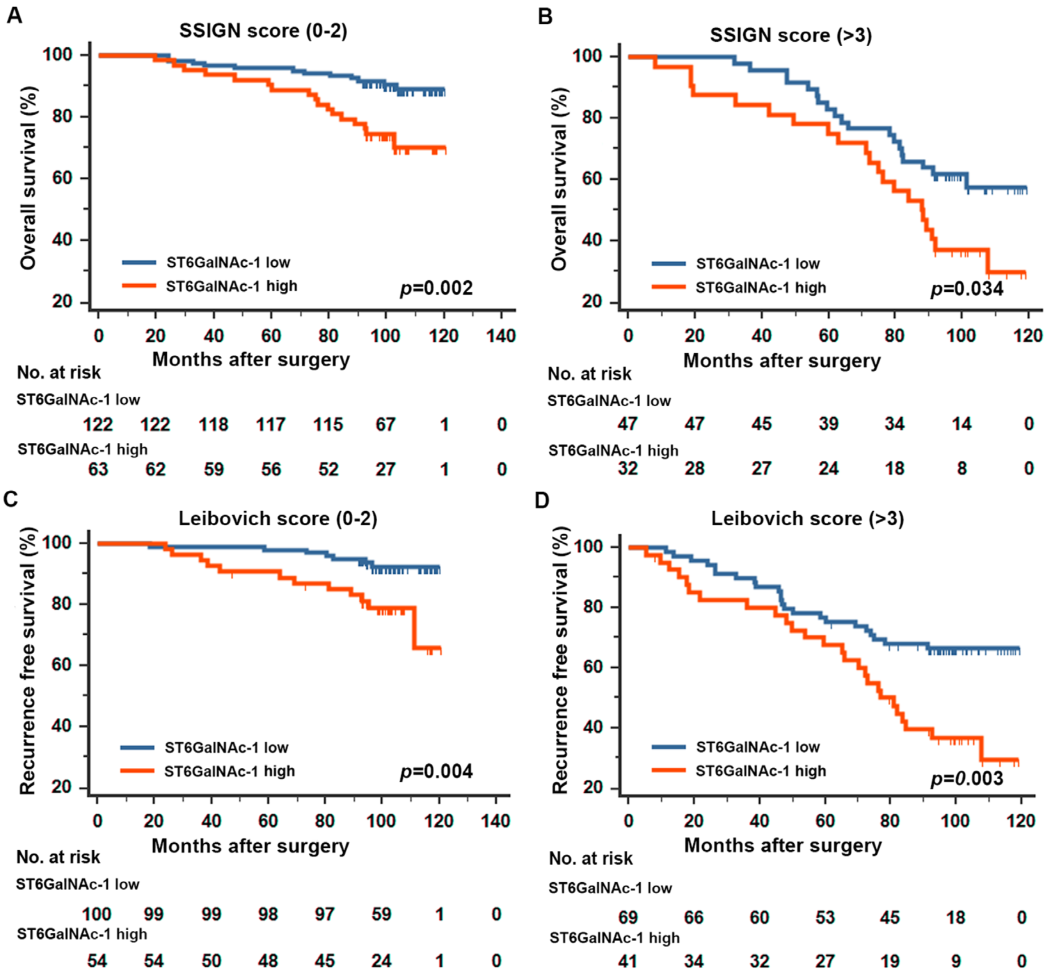

Supplementary Figure S1: Kaplan-Meier analyses for overall survival and recurrence free survival of patients in SSIGN/Leibovich subgroups. Overall survival for patients in the SSIGN low risk group **A.** and high risk group **B.** according to ST6GalNAc-1 expression; recurrence free survival for patients in the Leibovich low risk group **C.** and high risk group **D.** according to ST6GalNAc-1 expression; p-value was calculated by Log rank test, $p<0.05$ was regarded as statistically significant.

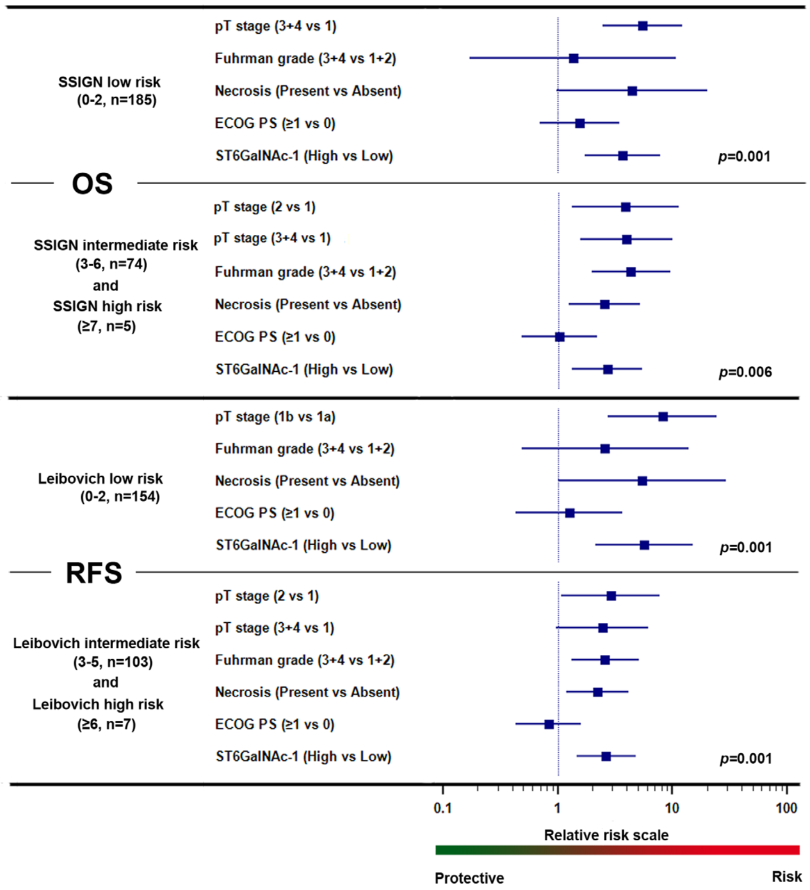

Supplementary Figure S2: Hazard ratios for overall survival and recurrence free survival probabilities based on ST6GalNAc-1 expression levels in different SSIGN/Leibovich subgroups.
